# Cognitive Behavioral Therapy Normalizes Functional Connectivity for Social Threat in Psychosis

**DOI:** 10.1093/schbul/sbv153

**Published:** 2015-10-27

**Authors:** Liam Mason, Emmanuelle R. Peters, Danai Dima, Steven C. Williams, Veena Kumari

**Affiliations:** ^1^Department of Psychology, Institute of Psychiatry, Psychology and Neuroscience, King’s College London, London, UK;; ^2^South London and Maudsley NHS Foundation Trust, NIHR Biomedical Research Centre for Mental Health, London, UK;; ^3^MRC Social Genetic and Developmental Psychiatry, Institute of Psychiatry, Psychology and Neuroscience, King’s College London, London, UK;; ^4^Psychosis Research Program, Department of Psychiatry, Icahn School of Medicine at Mount Sinai, New York, NY;; ^5^Department of Neuroimaging, Institute of Psychiatry, Psychology and Neuroscience, King’s College London, London, UK

**Keywords:** connectivity, CBT, therapy, psychosis, amygdala

## Abstract

Psychosis is often characterized by paranoia and poor social functioning. Neurally, there is evidence of functional dysconnectivity including abnormalities when processing facial affect. We sought to establish whether these abnormalities are resolved by cognitive behavioral therapy for psychosis (CBTp). The study involved 38 outpatients with one or more persistent positive psychotic symptoms, and 20 healthy participants. All participants completed an implicit facial affect processing task during functional magnetic resonance imaging (fMRI). Subsequently, patients either continued to receive standard care only (SCO, *n* = 16) or received CBTp on top of standard care (+CBTp, *n* = 22), with fMRI repeated 6–8 months later. To examine the mechanisms underlying CBTp-led changes in threat processing and appraisal, functional connectivity during the social threat (angry faces) condition was assessed separately from left amygdala and right dorsolateral prefrontal cortex (DLPFC) seeds. At baseline, patients, compared with healthy participants, showed greater amygdala connectivity with the insula and visual areas, but less connectivity with somatosensory areas. These differences normalized following CBTp and, compared with the SCO group, the +CBTp group showed greater increases in amygdala connectivity with DLPFC and inferior parietal lobule, with the latter correlating with improvement in positive symptoms. From the DLPFC seed, the +CBTp (compared with SCO) group showed significantly greater increase in DLPFC connectivity with other prefrontal regions including dorsal anterior cingulate and ventromedial prefrontal cortex. These findings indicate that CBTp strengthens connectivity between higher-order cognitive systems and those involved in threat and salience, potentially facilitating reappraisal.

## Introduction

There has been a growing interest in understanding the neural effects of psychological interventions, to gain a better understanding of the maintaining mechanisms of disorders and to improve future therapies. There is now considerable evidence that interventions such as a cognitive behavioral therapy produce lasting neural activation changes in a range of disorders.^1^ However, few to date have examined changes in the widespread connections between distributed systems. This is surprising, given that cognitive behavioral therapy targets dysfunctional appraisals of distributed cognitive, affective, and physical inputs.^2^ Remediation of functional connectivity is likely to be of particular importance in psychosis, which has long been conceptualized as a disorder of network dysconnectivity.^3,4^ Yet, to date, none have examined changes in functional connectivity following psychological therapy in psychosis.

Psychological models of psychosis emphasize a hyper-vigilance for threat cues as well as a difficulty in disengaging threat-related affective content from conscious awareness.^5,6^ Neurobiological accounts are in accordance with this view^7^ and there is convincing evidence of a bias to perceive facial expressions as threatening.^8^ This social threat network includes limbic (amygdala, putamen, thalamus), visual (fusiform gyrus), and prefrontal regions, in addition to the insula.^9^ In healthy participants, cognitive reappraisal strategies have been shown to modulate activity in this network, through top-down connections with frontal areas such as the dorsolateral prefrontal cortex (DLPFC).^10,11^ However, in psychosis there is reduced amygdala connectivity with prefrontal regions when processing threatening faces,^12–14^ as well as a reduced ability to regulate insula.^15–17^ At the symptom level, these abnormalities in social threat perception are likely to be involved in the generation and maintenance of persecutory delusions and poor social functioning.^18^ Accordingly these are an important therapeutic target.

There is considerable evidence that psychological interventions target DLPFC and other prefrontal regions,^1,19^ perhaps linking to a role in reappraisal of affective inputs mentioned previously.^10,11^ However, there are no studies to date that have examined changes in functional connectivity following psychological intervention in psychosis. In studies of social threat processing in anxiety disorders, functional connectivity of the amygdala has been implicated both as a predictor of,^20^ and a responder to,^21^ CBT. Furthermore, in psychosis, connectivity changes following treatment with antipsychotic medication frequently involve the DLPFC and parietal lobule, as well as other frontal and temporal areas.^22–24^ The inferior parietal lobule (IPL) is involved in empathy,^25^ self-awareness,^16,26^ and self-other discrimination.^27^ Consistent with these functions, IPL has been linked to cognitive insight; the ability to reflect on one’s own beliefs and to entertain those of others^16,28^ which is a predictor of favorable CBTp outcome.^29^

We have previously shown that cognitive behavioral therapy for psychosis (CBTp) brings about significant changes in the processing of social threat. Specifically, there was a reduction in the activation of arousal and salience regions including insula, thalamus, putamen, and visual areas, and these changes correlated with improvement in positive symptoms.^30^ The present study sought to elaborate on the mechanism underlying these functional activation changes by examining, in the same patients, concomitant changes in functional connectivity with higher-order cognitive systems. A further aim was to establish whether CBTp-led changes result in normalization of connectivity by additionally comparing connectivity with healthy participants. We hypothesized that patients with psychosis would show greater recruitment of affective and salience regions with reduced regulation from higher cognitive areas. In line with this, we predicted greater amygdala connectivity with insula and reduced connectivity with DLPFC^12–14^ and the parietal lobule,^22–24^ compared with healthy participants. We further predicted that these abnormalities would normalize following CBTp and that CBTp-led changes in connectivity would correlate with improvement in psychotic symptoms.

## Methods

### Participants and Design

Participants were 38 right-handed outpatients with a diagnosis of paranoid schizophrenia,^31^ and 20 healthy participants matched, on average, to patients for age, sex, handedness, and years of education (table 1). Patients were recruited from community services in the South London and Maudsley NHS Foundation Trust (SLaM). Creation of the study groups followed a cohort case-controlled design in which one inclusion criteria was that all included patients were deemed as suitable for CBTp by consultant psychiatrists. Hence receipt vs nonreceipt of CBTp was independent of the research team and resulted from clinical resource limitations of the NHS Trust, rather than patient characteristics. Participants receiving standard care only (SCO group) were matched to participants receiving CBTp on top of standard care (+CBTp group) on the basis of having similar clinical and demographic characteristics. All patients had been on stable doses of antipsychotic medication for at least 3 months at entry to the study (ie, at T1) and this was not changed during the study. Hence, only the +CBTp group received any additional intervention as part of the study. The study procedures were approved by the ethics committee of the joint research ethics committee of SLaM and Institute of Psychiatry, Psychology & Neuroscience in London (ref: 209/02). All participants provided written informed consent and were compensated for their time and travel.

**Table 1.** Demographics, Task Performance, and Clinical Characteristics of Participants

			+CBTp Group (*n* = 22, 18 Male)			SCO Group (*n* = 16, 14 Male)		Healthy Participants (*n* = 20, 15 Male)
			Mean (SD)			Mean (SD)		Mean (SD)
			Baseline	Follow-up		Baseline	Follow-up	Baseline
Age (years)			35.7 (7.82)			39.2 (9.37)		37.3 (12.5)
Education (years)			13.9 (3.26)			13.6 (1.71)		14.8 (3.0)
Predicted IQ^a^			109.4 (9.68)			106.6 (9.73)		115 (8.0)
Age at illness onset			24.8 (8.38)			25.8 (8.49)		
Duration of illness (years)			10.9 (7.70)			13.4 (10.2)		
Medication			Atypical antipsychotic (*n* = 20); combined atypical and typical (*n* = 2)			Atypical antipsychotic (*n* = 14); combined atypical and typical (*n* = 2)		
Chlorpromazine equivalent (mg)			543 (479.3)			448.9 (338.8)		
Gender Discrimination Accuracy (%)	Neutral		92.6 (10.8)	91.8 (13.1)		88.3 (10.8)	90.0 (14.6)	92.5 (18.3)
	Fear		90.5 (14.4)	91.4 (16.5)		87.9 (20.9)	87.9 (18.5)	92.7 (15.8)
	Anger		88.6 (15.2)	88.9 (14.2)		84.8 (16.1)	87.3 (19.1)	91.3 (19.4)
	Happy		94.7 (8.48)	93.3 (9.94)		92.4 (7.82)	90.8 (12.3)	94.5 (18.7)
Detection (%)	No face		93.4 (12.4)	91.5 (16.4)		93.8 (13.7)	92.5 (18.1)	93.8 (22.3)
PANSS^b^ symptoms								
*Positive symptoms*			18.1 (4.84)	14.9 (4.10)		*t*(21) = 3.90*	18.6 (3.20)	18.1 (3.30)^ns^
*Negative symptoms*			17.7 (4.23)	15.6 (4.29)		*t*(21) = 2.61*	19.1 (4.13)	20.3 (4.38)^ns^
*General Psychopathology*			33.5 (7.24)	28.6 (7.40)		*t*(21) = 2.97*	35.4 (4.41)	35.4 (6.49)^ns^
*Total symptoms*			69.3 (13.3)	59.0 (14.7)		*t*(21) = 3.72*	73.1 (9.28)	73.8 (11.8)^ns^
BDI^c^ symptoms			16.2 (8.3)^d^	11.5 (9.9)*^d^		*t*(19) = 2.19*	15.9 (10.4)	15.8 (12.1)^ns^

^a^National Adult Reading Test.^32^

^b^Positive and Negative Syndrome Scale.^33^

^c^Beck Depression Inventory.^34^

^d^Missing data for 1 participant.

*Significant symptom reduction (*P* < 0.05) at follow-up relative to baseline.

Diagnosis was confirmed by means of the Structured Clinical Interview for DSM-IV (SCID).^31^ Psychotic symptoms were assessed blindly at both time points using the Positive and Negative Syndrome Scale (PANSS).^33^ Mood symptoms were assessed using the Beck Depression Inventory-II.^34^ All participants underwent functional magnetic resonance imaging (fMRI) at baseline (T1). Patients were scanned a second time 6–8 months later (T2), following either CBTp in addition to standard care (+CBTp group, *n* = 22) or standard care only (SCO group, *n* = 16). Healthy participants were recruited from the local community using existing research databases. Exclusion criteria included a history of mental illness, drug and alcohol abuse or a regular medical prescription. The absence of a clinical diagnosis in healthy participants was confirmed using the SCID nonpatient edition.^35^

### Task

In the scanner, participants performed an implicit facial affect task in which they were presented with monochrome faces depicting fear, anger, happiness, or neutral expressions.^36^ These 4 conditions were counterbalanced across 16 blocks lasting 30 s (8 faces per block, with 3.75 s per face). Each block was followed by a 15 s block containing 4 baseline trials in which an empty oval frame without the faces (but matched for luminance to the faces) was shown. On presentation of each face, participants had to indicate whether the face was male or female by pressing the left (female) or the right (male) button on a button box. During the baseline blocks, they were required to press either the left or the right button when the empty oval frame appeared. All participants practiced the gender discrimination task once on all identities on a laptop computer and an identical button box in advance of their scheduled fMRI scan.

### Cognitive Behavioral Therapy for Psychosis and Standard Care Procedures

The +CBTp group received 6–8 months of manualized CBT for psychosis^37^ (see supplementary material).

### Image Acquisition

Two hundred and forty T2*-weighted images were acquired using a 1.5 Tesla General Electric Signa system with the following parameters: echo time 40ms, repetition time 3 s, flip angle 90°, field of view 240mm, slice thickness 7.0mm, and interslice gap 0.7mm. In the same session, a high-resolution structural scan (T1-weighted images in the axial plane with 1.5mm contiguous sections) was acquired using a 3D inversion recovery prepared spoiled gradient recalled acquisition (echo time 5.1ms, repetition time 18ms, inversion time 450ms, flip angle 20 degrees with one data average, and 256×256×128 voxel matrix).

### Data Analysis

#### Sample Characteristics and Task Performance.

Analysis of variance (ANOVA) was used to compare the patient and healthy participant groups on their age, predicted IQ,^32^ and years of education. For patient groups, duration of illness, age of onset, and number of episodes were also compared. For task performance, the percentage of correct trials for gender discrimination of facial expressions was compared across the 3 groups at baseline (T1) and across the 2 patient groups that completed the task a second time (at T2) using ANOVA. For T1, the factors were group (healthy participants, SCO, +CBT) and condition (anger, happy, fear, neutral). This ANOVA was repeated for T2 including only the SCO and +CBT groups.

#### CBTp-led Changes in Symptoms and Task-Related Activations.

The analysis of CBT-led changes in symptoms and task-related neural activation patterns have been reported in full elsewhere^30^ and as such are summarized in the results section.

#### Functional Connectivity Analysis.

We assessed functional amygdala and DLPFC connectivity through the psycho-physiological interaction (PPI) approach^38^—see supplementary methods for more information.

#### CBTp-led Changes in Connectivity.

The main analyses focused on direct social threat (angry faces) condition, as this yielded the highest neural activation and was responsive to CBTp in previous work.^30^ The prosocial affect (happy faces) condition was also deemed to be of clinical importance given that patients with psychosis often misread prosocial gestures,^39^ potentially in line with cognitive biases which are likely to be amenable to CBTp. These analyses are reported as supplementary material due to space constraints.

Separate voxel-wise PPI analyses yielded statistical parametric maps containing positive connectivity coefficients for healthy (T1 only) and patient groups (both T1 and T2). After establishing that the 2 psychosis groups did not differ from each other at baseline, we established baseline abnormalities by comparing healthy participants to the patients (collapsed across groups) by using independent-samples *t*-tests within a random-effects analysis. Next, we assessed longitudinal changes in the psychosis groups from T1 to T2 by entering the connectivity maps into a random-effects ANOVA with factors group (+CBT, SCO) and time (T1, T2). We focused on changes in the +CBT group and examined the group–time interaction to establish significant group differences over time. Finally, we assessed whether the baseline abnormalities (T1) were still present at T2 by comparing both clinical groups with the healthy group (reported as supplementary material). Based on previous reports of CBT effects in other disorders,^20,40,41^ we expected that connectivity changes would be relatively subtle. We thus initially applied a threshold of uncorrected *P* < .001 (≥7 contiguous voxels) for all contrasts of interest. We then examined which findings survived more conservative family-wise error correction.

Finally, we examined whether the connectivity abnormalities observed at T1 were still present at T2, by comparing the +CBTp and SCO groups separately to the healthy group. The F-contrast “patients different from healthy participants” from T1 was used to functionally mask the contrasts at T2 (“+CBTp T2 different from healthy participants”; “SCO T2 different from healthy participants”). To reduce false negatives a more lenient threshold (uncorrected *P* < .05) was used to define the T1 functional mask (with our standard threshold of uncorrected *P* < .001 kept for the T2 contrast).

#### Correlation with Symptoms and Assessing Normalization.

After identifying areas of connectivity that responded to CBTp, we examined associations with symptom change on the PANSS in the +CBTp group. To do this, we first defined functional regions of interest (ROI) as 2mm spheres around the +CBTp group peaks of the connectivity changes (within-group and interactive effects). Next, we used MarsBar^42^ to extract the mean connectivity coefficients within these ROIs for both time points. We then explored associations between the CBTp-led connectivity changes and symptom improvement. To reduce the risk of type 1 error due to multiple statistical tests and to test for effects at the multivariate level, we performed multivariate analysis of variance (MANOVA) instead of multiple univariate analyses.^43^ The CBTp-led connectivity changes were entered into a MANOVA as separate dependent variables and the changes on the positive and negative subscales of the PANSS were entered as additional regressors, separately for the 2 seeds. The direction of any significant effects was determined by partial correlation tests (uncorrected).

## Results

### Sample Characteristics and Baseline Task Performance

Sample characteristics for the 2 patient groups and the healthy group are reported in table 1. At baseline, there were no differences between groups in terms of age, sex, or years of education (*P* ≥ .39). There were differences in predicted IQ [*F*(2, 54) = 4.69, *P* = .013], with the healthy participants showing higher scores than the SCO [*t*(54) = 2.22, *P* = .03] and +CBT [*t*(54) = 2.93, *P* = .005] groups but no difference between the 2 patient groups (*P* = .37). There were also no differences between the patient groups in terms of duration of illness, age of onset, number of episodes, or rates of drug use (*P* ≥ .19). Groups were also equivalent in terms of psychotic and depressive symptoms (*P* ≥ .32) and did not differ in their task performance for any condition at T1 (*P* ≥ .49) or at T2 (*P* ≥ .53).

### CBTp-led Changes in Symptoms and Task-Related Activations

Only the +CBTp group showed a significant improvement of symptoms at T2, as measured by PANSS and Beck Depression Inventory-II (table 1). Neurally, compared with SCO, the +CBTp group showed significant reductions in activation (from T1 to T2) in left inferior frontal gyrus, left insula, bilateral putamen, and left occipital areas (table 3 in ref.^30^). In addition, these changes correlated with improvement in symptoms of psychosis (table 5 in ref.^30^).

### Functional Connectivity Abnormalities in Patients at Baseline (T1)

Healthy participants showed positive amygdala connectivity with several frontal areas, including right frontopolar cortex, right anterior cingulated, and left premotor cortex, as well as with left precuneus and thalamus (supplementary table 1). Compared to healthy participants, patients (collapsed across groups) showed greater amygdala connectivity with right lingual gyrus and left insula (straddling anterior and posterior segments), but less connectivity with left postcentral gyrus (table 2; see supplementary table 2 for network at T1 in psychosis participants).

**Table 2.** Group Differences in Baseline (T1) Connectivity from Amygdala for Angry Faces Condition

Contrast	MNI Coordinates			*Z* Score	Cluster Size	Brodmann Area	Region
	*x*	*y*	*z*				
AMYG							
Healthy > Patients	44	−16	28	3.1	7	3	Postcentral gyrus
Patients > Healthy	32	−62	10	3.12	10	19/37	Lingual gyrus
	−44	−10	22	3.06	1 (10*)	13	Insula

*Uncorrected *P* < .005.

From the DLPFC seed, healthy participants showed widespread connectivity, including right insula, bilateral inferior frontal gyrus, left premotor cortex, right visual cortex, right superior parietal lobule, as well as mid- and superior- temporal areas bilaterally (supplementary table 1). Similar connections were observed in the psychosis participants, although there were no cingulate or precentral gyrus connections (supplementary table 2). However, differences between the healthy and psychosis participants did not reach significance at this time point.

### Longitudinal Changes in Functional Connectivity (from T1 to T2)

There were no differences between the +CBT and SCO groups at baseline for either the amygdala or DLPFC seeded connectivity analyses at our voxel threshold (uncorrected *P* < .001).

**Fig. 1.**
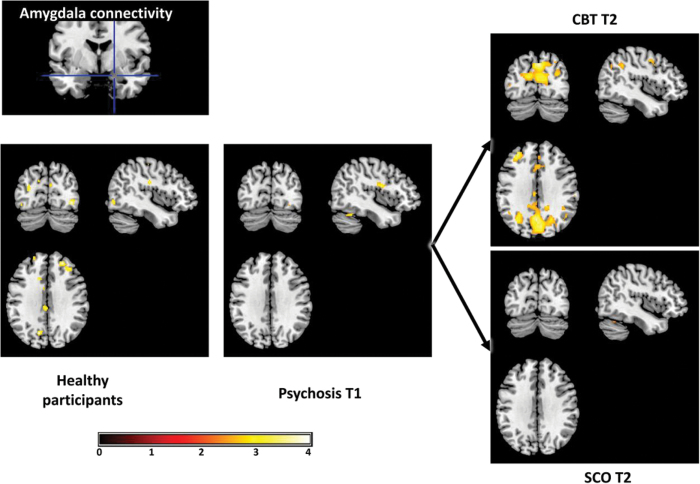
Cognitive behavioral therapy for psychosis (CBTp) increases threat-related amygdala connectivity. At baseline (T1), the prefrontal and visual cortical connectivity evident in healthy participants (far left) were not evident in the psychosis participants. There were post-therapy increases in connectivity in these connections, as well as the inferior parietal lobule, in the +CBTp but not SCO group. Visualized with voxel threshold of *P* < .005 (uncorrected) around centre *x* = −43, *y* = −76, *z* = 33.

**Fig. 2.**
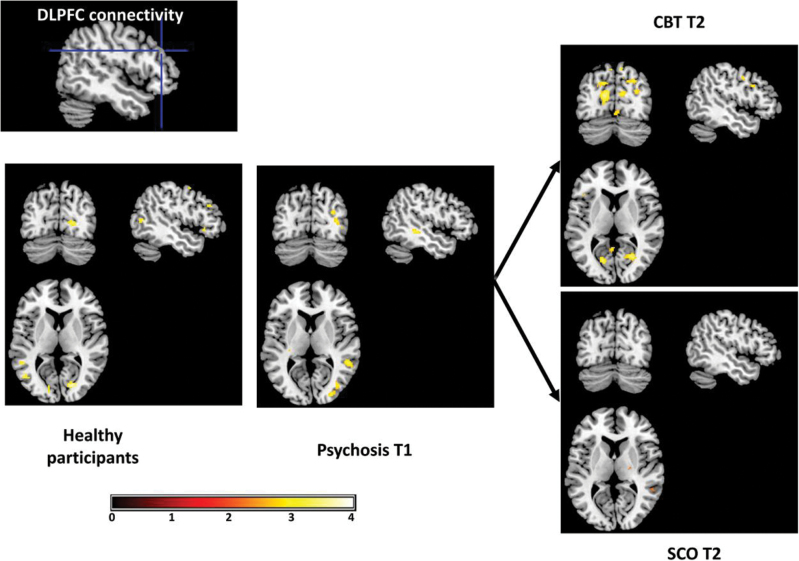
Cognitive behavioral therapy for psychosis (CBTp) increases threat-related dorsolateral prefrontal cortical connectivity. There were post-therapy increases in connectivity with visual, prefrontal, and cingulate cortices in the +CBTp but not SCO group. Visualized with voxel threshold of *P* < .005 (uncorrected) around centre *x* = −49, *y* = −73, *z* = 8.

Within the +CBTp group, there were T1 to T2 increases in amygdala connectivity with left DLPFC and right IPL (figure 1; supplementary table 3) as well as bilateral premotor cortex and posterior cingulate gyrus. There were no reductions in connectivity in this group. To examine significant group differences in connectivity changes, we tested for group–time interaction effects. Compared with the SCO group, the +CBT group showed significantly greater increases in amygdala connectivity with right DLPFC, IPL, and posterior cingulate gyrus as well as left SPL, postcentral gyrus, and thalamus (figure 2, left pane). They also showed a greater reduction in connectivity with a cluster bordering left insula and inferior frontal gyrus.

**Fig. 3.**
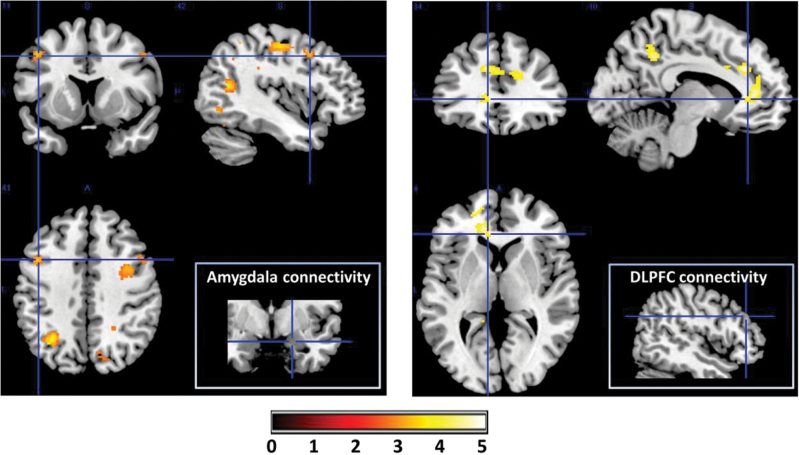
Cognitive behavioral therapy for psychosis (CBTp) increases functional connectivity for processing social threat. Compared with the standard care control group, the +CBTp group showed significantly greater increases in amygdala connectivity with a range of regions, including dorsolateral prefrontal cortex (DLPFC) and the inferior parietal lobule (left). Similarly the +CBTp group showed greater increases in DLPFC connectivity with other executive control regions, including dorsal (and ventral, not shown) portions of the anterior cingulate (right). Group-by-time interaction visualized with voxel threshold of *P* < .005 (uncorrected) around crosshair centres: amygdala (*x* = −43, *y* = 11, *z* = 41] and DLPFC (*x* = −10, *y* = 34, *z* = 4).

From the DLPFC seed, the +CBTp group showed increased connectivity with left visual cortex (from T1 to T2; figure 3; supplementary table 4). The +CBT group showed significantly greater increases in DLPFC connectivity with dorsal and subgenual portions of the left anterior cingulate cortex and with left posterior cingulate, extending into the IPL (figure 2, right pane).

Finally, none of the above findings survived family-wise error correction.

### Functional Connectivity Associations with Symptom Change

**Fig. 4.**
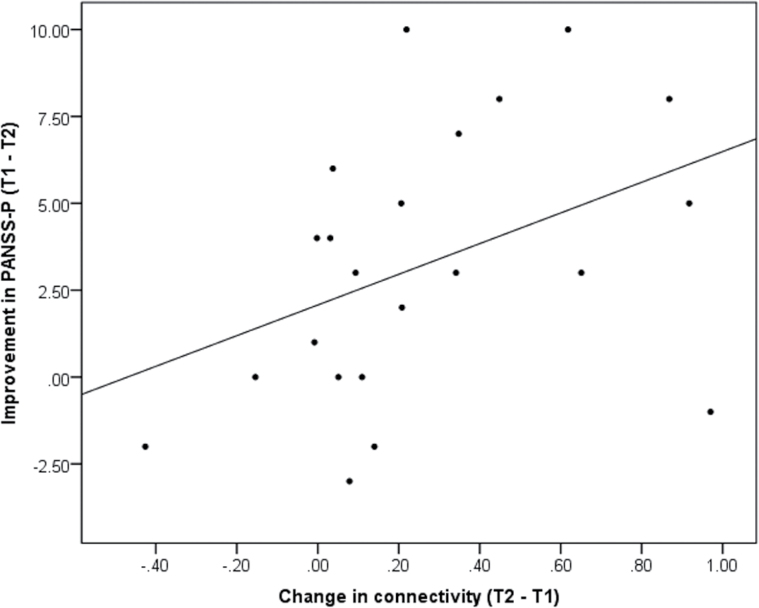
Increase in amygdala connectivity with inferior parietal lobule following cognitive behavioral therapy for psychosis correlates with improvement in positive symptoms of psychosis (PANSS-P). Connectivity scale is in beta values.

For the amygdala seed, the improvement in positive psychotic symptom (PANSS-P) marginally covaried with the change connectivity with IPL [*F*(1, 19) = 3.28, *P* = .08; positive correlation: *r*(22) = .43, *P* = .05; figure 4] and SPL [*F*(1, 19) = 3.66, *P* = .07; positive correlation: *r*(22) = .4, *P* = .07]. There were no other univariate effects for either the amygdala (*P* ≥ .1) or DLPFC (*P* ≥ .11) models.

To better understand the relationship with symptoms, these effects were followed up with correlations with improvement on individual PANSS-P items. The increase in connectivity with IPL was specifically and positively correlated with improvement on the perceived persecution (PANSS-P6) item [Spearman’s ρ(22) = .66, *P* = .001] but no other items, either for IPL (*P* ≥ .26) or for SPL (*P* ≥ .27) connections.

### Functional Connectivity Abnormalities Revisited at T2

Further analyses examined whether the connectivity abnormalities observed at T1 were still present at T2, in the +CBTp and SCO groups separately. None of the 3 baseline amygdala abnormalities remained in the +CBTp group when compared again to the healthy group at T2. In contrast, the SCO group continued to show dysconnectivity with somatosensory cortex and, while dysconnectivity with insula was no longer significant at our a priori statistical threshold, it remained at a less conservative threshold (uncorrected *P* = .008). Furthermore, normalization of these abnormalities correlated with the CBTp-led changes in amygdala connectivity with DLPFC (see supplementary materials).

## Discussion

This study found that CBTp resulted in reorganization of brain networks involved in processing social threat.

At baseline, psychosis patients showed amygdala hyperconnectivity with insula and visual occipital areas, compared with healthy participants. These findings are consistent with theoretical accounts of inappropriate activation of the salience network in psychosis, which may underlie hyper-vigilance to social threat and susceptibility to paranoia.^17^ The psychosis groups additionally showed reduced connectivity between amygdala and somatosensory areas. This finding is consistent with previous reports in psychosis of reduced activation in somatosensory areas when processing facial affect, which is associated with social anhedonia.^44^ Although not predicted, this is in line with previous reports of reduced activation in somatosensory areas when processing facial affect and can be explained by the role of the somatosensory cortex in supporting recognition of facial affect by providing a visuo-somatic reference template.^45^ Hence, reduced coupling between amygdala and somatosensory cortex may indicate that when engaging in facial affect discrimination, people with psychosis do not optimally integrate affective and perceptual aspects, a finding which may explain misperception of affect.^18^ Importantly, there was evidence that these baseline differences normalized following CBTp, whereas the group receiving SCO continued to show amygdala dysconnectivity with somatosensory areas, compared with the healthy group. This CBTp-specific normalization tallies with reports that social skills training interventions result in increased somatosensory activation during facial affect recognition.^46,47^

In addition to normalization, there was additionally a widespread proliferation in connectivity post CBTp, as evidenced by changes in the number and strength of connections from amygdala and DLPFC (from T1 to T2). This diverse proliferation of functional connections is evidence that CBTp promotes integration of multiple and distributed neural systems, which may be particularly relevant for psychosis as a dysconnectivity syndrome.^3^

Many of these connections occurred within the threat network, including amygdala, inferior frontal gyrus, thalamus, and visual areas, which had, in previous analyses, shown reduced activation following CBTp.^30^ Together, these analyses better explicate a therapeutic mechanism in which aberrant threat network activation is reduced following CBTp, perhaps especially through improved top-down regulation from prefrontal cortical regions. There was also evidence of greater connectivity within prefrontal cortical networks, as evidenced by greater DLPFC connectivity with dorsal anterior cingulate cortex, involved in cognitive control,^48^ as well as ventromedial prefrontal cortex, which may act as a gateway for regulatory DLPFC projections to amygdala.^49^ Hence, CBTp may reduce threatening appraisals by integrating affective and salience signals with higher cognitive processes, such as reappraisal.^11^ Although not tested here, this explanation is in line with the aims of CBTp to shape cognitive appraisals of anomalous experiences and to regulate affective disturbances.^6,37^

Although no other studies have examined changes in connectivity following psychological therapy for psychosis, there is initial evidence of increased DLPFC regulation of amygdala during facial affective processing in social anxiety disorder.^21^ Further work will be needed to examine the possibility that this represents a transdiagnostic therapeutic mechanism for social threat.

Also of clinical importance were the CBTp-specific increases in amygdala connectivity with IPL, which correlated with improvement in persecutory beliefs (measured by the positive symptoms scale of the PANSS). This may be attributable to the role of the IPL in self-other discrimination generally^27^ as well as clinical and cognitive insight in psychosis.^16,28^ There is evidence of increased (superior) parietal lobule connectivity following pharmacotherapy with antipsychotics,^22^ highlighting a common outcome irrespective of treatment modality. Further work will be needed to probe the potential overlap in neural mechanisms.

A limitation of the current study design is that the groups were not randomized and so we cannot rule out effects of selection bias for take-up of CBTp. In addition, healthy participants were only tested once and so our analyses to determine normalization did not take into account potential practice effects that could have occurred in the healthy participants had they been tested a second time. The present results did not survive family-wise error correction, increasing the possibility of type 1 errors. It should be noted however that the statistical threshold we applied is at least equal to, if not more conservative than, all extant reports of connectivity changes following CBT in other disorders.^20,40,41^ Collectively, the research indicates that the changes are relatively subtle or not well captured by existing data capture and/or analysis methods.

In summary, this study provides evidence that CBTp leads to reorganization of amygdala and prefrontal cortex connections mediating social threat processing. These changes included, but go beyond, normalization of the pathophysiology detected at baseline and include additional, compensatory changes. Only a single (prefronto-parietal cortical) connection correlated with improvement in psychotic symptoms, highlighting that additional, more sophisticated clinical psychometrics are needed to better capture the psychoneurobiological changes following CBTp.

## Supplementary Material

Supplementary material is available at http://schizophreniabulletin.oxfordjournals.org.

## Funding

The Wellcome Trust (067427/z/02/z SRF to V.K.); Biomedical Research Centre for Mental Health at the Institute of Psychiatry, Psychology & Neuroscience (King’s College London) and South London and Maudsley NHS Foundation Trust, UK (partial support to V.K.).

## Supplementary Material

Supplementary Data
