# Supplementary material for: Cognitive Behavioral Therapy Normalizes Functional Connectivity for Social Threat in Psychosis
Source: Schizophr Bull. 2015 Oct 27;42(3):684–92. doi: 10.1093/schbul/sbv153 (PMC4838085; doi:10.1093/schbul/sbv153)
Supplement: Supplementary Data [file supp_42_3_684__index.html]

Cognitive Behavioral Therapy Normalizes Functional Connectivity for Social Threat in Psychosis — Supplementary Data 

# Cognitive Behavioral Therapy Normalizes Functional Connectivity for Social Threat in Psychosis

## Supplementary Data

Data files

- Supplementary Data - Supplementary Data
