# Supplementary material for: Cognitive Behavioral Therapy Normalizes Functional Connectivity for Social Threat in Psychosis
Source: Schizophr Bull. 2015 Oct 27;42(3):684–92. doi: 10.1093/schbul/sbv153 (PMC4838085; doi:10.1093/schbul/sbv153)
Supplement: Supplementary Data [file supp_sbv153_Supplementary_Materials_R1.doc]

**SUPPLEMENTARY MATERIALS**

**Methods**

**Cognitive behavioural therapy for psychosis and standard care procedures**

Patients received approximately 16 sessions which took place weekly or fortnightly at the patient’s preference. CBTp was formulation driven and focused on the goals of the patient. Therapists received supervision from clinical psychologists with extensive experienced in the intervention. Treatment adherence was recorded in supervision and a small selection of therapy sessions (n = 13) were also selected, at random, for rating from an independent therapist. The ongoing standard care (received by both the +CBTp and SCO groups) consisted of community case management with regular care coordination (e.g. from a community psychiatric nurse) and access to psychiatry and other clinical input as needed.

**CBTp-led changes in symptoms and task-related activations**

Briefly stated, the change in symptoms was examined by a Group (SCO, +CBTp) x Time (T1, T2) ANOVA with Group as the between-subjects factor and Time as the within-subjects factor, and then a Group x Time effect was followed-up by paired t-tests on total and sub-scale PANSS scores separately in the +CBTp and SCO groups. Preprocessing and analysis of fMRI data to determine task and CBTp effects was performed using statistical parametric mapping software (SPM5; http://www.fil.ion.ucl.ac.uk/spm). During pre-processing, for each participant, the 240 volume functional time series were motion corrected, transformed into stereotactic space (Montreal Neurological Institute, MNI), smoothed with a 8 mm FWHM Gaussian filter and band pass filtered. The first-level analysis involved modelling the fMRI responses using a canonical haemodynamic response function convolved with the vectors of interest. For each participant, the four facial affect conditions were entered into the design matrix as explanatory variables, with the no face condition serving as an implicit baseline. Six movement parameter vectors (translation x, y, z and rotation x, y, z) generated from the realignment procedure were also modelled as regressors of no interest. The model additionally included drift terms up to 1/128 Hz to remove low frequency components, and global confounds were removed using global normalisation. Generic task-related activations were identified for each facial expression condition using one-sample t-tests. Group-level analyses were based on random-effects analyses of the single-subject contrast images.

**Functional connectivity analysis**

This consists of a design matrix of three regressors: the experimental task (the “psychological variable”); the activation time course for the seed region (“physiological variable”); and a variable representing their interaction. We defined two seeds from the group-level maxima identified by prior activation analyses. For amygdala we took the peak voxel in right amygdala for the Anger > Baseline contrast at baseline (x=20, y=-2, z=-18, Kumari et al., 2011; T = 5.91). For DLPFC, this region was not identified by activation analyses but was theorised to be important based on previous work examining the mechanisms of CBT and affective face processing . We defined this seed from the peak voxel in left DLPFC (x=48, y=34, z=30; T = 4.43), which predicted response to CBT in an overlapping cohort performing a working memory task (2 back > 0 back) [^40^](#_ENREF_40). For both seeds, we chose the hemisphere with the greatest activation, although there was no significant difference in activation by hemisphere for either structure. Regions of interest were created from spheres around the peak voxels (of 3 mm and 4 mm radius for amygdala and DLPFC respectively), anatomically constrained using definitions from the PickAtlas toolbox [^41^](#_ENREF_41).

**Results**

**Indirect CBTp-led influence on baseline abnormalities (active threat condition)**

We examined whether normalisation of the baseline markers was related to the CBTp-led changes in connectivity (see Main Results). A multivariate analysis was performed with change in the CBTp-responsive connections (IPL and DLPFC) as separate dependent variables, normalisation of the connectivity abnormalities (lingual gyrus, insula and somatosensory cortex) as separate covariates and group as a fixed factor. Two of the baseline abnormalities exerted significant multivariate effects in the model [somatosensory area: F(2,32) = 7.26, p = .002; lingual gyrus: F(2,32) = 3.41, p = .046], as did group F(2,32) = 6.28, p = .005], whereas insula did not reach significance (p = .1). Within the model, the change in the baseline somatosensory abnormality covaried with the CBTp-led change in IPL [F(1, 33) = 13.0, p < .001] and marginally in DLPFC [F(1, 33) = 3.59, p = .06]. Similarly the change in the baseline lingual gyrus abnormality covaried with the CBTp-led change in DLPFC [F(1, 33) = 5.34, p = .028] whereas the IPL covariate did not reach significance (p = .11). Finally the change in the baseline insula abnormality marginally covaried with the CBTp-led change for DLPFC [F(1, 32) = 3.64, p = .06] but not IPL (p = .17). Correlations confirmed that the directionality of these effects were all in the positive direction (r ≥ .35).

**Comparison of direct and indirect social threat**

Both control and psychosis participants showed the strongest effects in the anger condition (reported in Main Results). Findings were highly comparable for the fearful condition (data available upon request). Of note, the psychosis participants showing at baseline less amygdala connectivity with left IPL which, importantly, normalised in only the +CBTp group (who showed increases in amygdala connectivity with left IPL at follow-up compared to baseline, as per findings for the direct threat condition). There were no areas that increased their connectivity with DLPFC following CBTp.

**Prosocial (non-threatening) affect**

**Group effects.**

For the happy condition the psychosis participants showed, at baseline, weaker amygdala connectivity with inferior parietal lobule, posterior cingulate, premotor cortex, inferior frontal gyrus, as well as dorsolateral and dorsomedial areas of prefrontal cortex (but no areas of stronger connectivity; Supplementary Table 5). In terms of the amygdala connectivity changes from baseline to follow-up, there were no significant differences between the +CBTp and SCO groups.

From the DLPFC seed, the psychosis participants showed weaker connectivity with dorsal anterior cingulate and a cluster bordering somatosensory and motor areas (and no areas of stronger connectivity; Supplementary Table 5). From baseline to follow-up (Supplementary Table 7), the +CBTp group showed significantly greater changes in connectivity with thalamus as well as somatosensory and superior temporal areas, compared to the SCO group.

**Associations with symptom change.**

There were no effects for the amygdala seed (p ≥ .17). For the DLPFC seed, PANSS-P improvement covaried marginally with the change in connectivity with postcentral gyrus [F(1, 19) = 4.24, p = .054; negative partial correlation: r(19) = -.43, p = .054] and marginally with thalamus connectivity [F(1, 19) = 3.49, p = .077; negative partial correlation: r(19) = -.39, p = .077]. PANSS-N improvement covaried with the change in connectivity with thalamus [F(1, 19) = 6.3, p = .021; positive partial correlation: r(19) = .499, p = .02] and parahippocampal gyrus [F(1, 19) = 5.19, p = .035; positive partial correlation: r(19) = .46, p = .035]. Associations with individual symptom items can be found in Supplementary Tables 8 and 9.

**Discussion**

**Normalisation of baseline connectivity abnormalities for direct threat**

The normalisation of baseline abnormalities (identified by contrasting the psychosis participants with healthy participants; see Main Discussion) covaried with change in the CBTp-responsive connections (IPL and DLPFC), suggesting that changes to the baseline abnormalities may be secondary to the main CBTp-led changes. In this way the normalisation of affective-salience hyperconnectivity (amygdala and insula) may be a ‘knock-on’ effect of better cognitive regulation of affective arousal (amygdala connectivity with DLPFC) and greater integration with self-awareness and cognitive insight (amygdala connectivity with IPL; see Main Discussion). Further work will be needed to establish this possibility and confirm the directionality.

**Connectivity for processing of prosocial social cues**

As with the direct threat condition, the psychosis groups showed, at baseline, reduced amygdala connectivity with prefrontal areas involved in higher cognition (e.g. DLPFC) and somatosensory empathy and motor preparation (somatosensory cortex; see Main Discussion), in addition to social cognition (IPL). Together, these findings indicate a global deficit in social affective processing, regardless of stimuli valence. As with the direct threat condition however, there were increases in amygdala connectivity with premotor cortex and with visual areas following CBTp. These findings may indicate greater engagement and processing of stimuli and improved integration with social cognition areas (see also changes in IPL connectivity with DLPFC, below).

The psychosis groups additionally showed, at baseline, reduced DLPFC connectivity with a region bordering somatosensory and motor cortices, most likely indicating a reduced cognitive priming of approach behaviour. Importantly, the reduced DLPFC connectivity with premotor cortex normalised following CBTp, potentially indicating a greater tendency to approach prosocial social cues following treatment. prosocialprosocial

**References**

1. Stephan K, Magnotta V, White T, Arndt S, Flaum M, O'LEARY D, et al. (2001): Effects of olanzapine on cerebellar functional connectivity in schizophrenia measured by fMRI during a simple motor task. *Psychological medicine*. 31:1065-1078.

Supplementary Table 1. Connectivity from amygdala and dorsolateral prefrontal cortex (DLPFC) seeds in healthy participants (direct social threat condition). Voxel threshold of uncorrected p < .001, except for specified *a priori* regions.

| **Seed** | **MNI coordinates** | | | **Z score** | **Cluster  size** | **Brodmann Area** | **Region** |
| --- | --- | --- | --- | --- | --- | --- | --- |
|  | **x** | **y** | **z** |  |  |  |  |
| *Amygdala* |  |  |  |  |  |  |  |
|  | -6 | 4 | 40 | 3.56 | 19 | 24 | Ventral anterior cingulate |
|  | -14 | -70 | 34 | 3.53 | 13 | 32 | Precuneus |
|  | 10 | 38 | 14 | 3.53 | 29 | 32 | Dorsal anterior cingulate |
|  | 24 | 44 | 32 | 3.52 | 16 | 46/9 | DLPFC / DMPFC |
|  | 38 | -76 | -6 | 3.47 | 11 | 19 | Extrastriate / MOG |
|  | -36 | 4 | 46 | 3.46 | 8 | 6 | Premotor cortex |
|  | -12 | -10 | 0 | 3.43 | 6 | *n/a* | Thalamus |
| *DLPFC* |  |  |  |  |  |  |  |
|  | 22 | -76 | 4 | 3.92 | 52 | 18 | Cuneus |
|  | -42 | 8 | 54 | 3.59 | 24 | 6 | Premotor cortex |
|  | -52 | 30 | 30 | 3.57 | 11 | 46 | DLPFC |
|  | 18 | -68 | 64 | 3.53 | 23 | 7 | Precuneus |
|  | 16 | 30 | 14 | 3.31 | 9 | 25/11 | Ventral anterior cingulate / orbitofrontal cortex |
|  |  |  |  |  |  |  |  |

Supplementary Table 2. Connectivity from amygdala and dorsolateral prefrontal cortex (DLPFC) seeds in all psychosis participants (direct social threat condition). Voxel threshold of uncorrected p < .001.

| **Seed** | **MNI coordinates** | | | **Z score** | **Cluster  size** | **Brodmann Area** | **Region** |
| --- | --- | --- | --- | --- | --- | --- | --- |
|  | **x** | **y** | **z** |  |  |  |  |
| *Amygdala* |  |  |  |  |  |  |  |
|  | 18 | -22 | -8 | 3.43 | 27 | n/a | Thalamus |
|  | 40 | -54 | -28 | 3.41 | 13 | n/a | Culmen |
|  | 42 | -4 | 22 | 3.31 | 5 | 13 | Insula/Precentral gyrus |
| *DLPFC* |  |  |  |  |  |  |  |
|  | 38 | -32 | 30 | 3.65 | 71 | 2/40 | IPL |
|  | 42 | -40 | -12 | 3.39 | 8 | 37 | Fusiform gyrus |
|  | -26 | -24 | 18 | 3.31 | 10 | 13 | Posterior insula |
|  | -50 | -32 | -4 | 3.3 | 9 | 21/22 | MTG/STG |
|  | -10 | -84 | 22 | 3.19 | 6 | 18/19 | Cuneus: SOG/MOG |

Supplementary Table 3 Differences in amygdala connectivity between baseline (T1) to follow-up (T2) in the cognitive behavioural therapy for psychosis (+CBTp) compared to standard care only (SCO) group for direct social threat (angry faces) condition. Voxel threshold of uncorrected p = .001.

| **Contrast** | **MNI coordinates** | | | **Z score** |  | **Brodmann Area** |  |
| --- | --- | --- | --- | --- | --- | --- | --- |
|  | **X** | **y** | **z** |  | **Cluster size** |  | **Region** |
| *+CBTp*  *T1 > T2* | *Nil* |  |  |  |  |  |  |
|  |  |  |  |  |  |  |  |
| *+CBTp*  *T2 > T1* |  |  |  |  |  |  |  |
|  | 34 | -4 | 48 | 3.95 | 38 | 6 | Premotor cortex |
|  | 18 | -42 | 28 | 3.88 | 24 | 31 | Posterior cingulate gyrus |
|  | 60 | -24 | 32 | 3.52 | 20 | 40 | Inferior parietal lobule |
|  | -6 | -26 | 24 | 3.42 | 18 | 23 | Posterior cingulate gyrus |
|  | -22 | -12 | 40 | 3.41 | 12 | 6 | Premotor cortex |
|  | -50 | 10 | 30 | 3.17 | 7 | 44 | DLPFC |
|  |  |  |  |  |  |  |  |
| CBT[T2>T1] > SCO[T2>T1] | 20 | -44 | 30 | 4.62 | 659 | 31 | Posterior cingulate gyrus |
|  | *6* | *-26* | *24* |  |  |  |  |
|  | *-4* | *-26* | *24* |  |  |  |  |
|  | 34 | -4 | 48 | 4.25 | 92 | 6/44 | Premotor cortex extends into DLPFC |
|  | -34 | -62 | 38 | 3s.78 | 112 | 7/39 | SPL: intraparietal sulcus /angular gyrus |
|  | -46 | -12 | 50 | 3.61 | 61 | 4/6 | Precentral gyrus / premotor cortex |
|  | -24 | -26 | 48 | 3.59 | 33 | 4 | Motor cortex |
|  | 60 | -28 | 36 | 3.58 | 30 | 2/40 | Postcentral gyrus/IPL |
|  | -34 | -56 | 54 | 3.46 | 8 | 7/40 | SPL |
|  | -42 | -60 | 14 | 3.4 | 22 | 37 | Occipitotemporal area / IPL: angular gyrus |
|  | -36 | -40 | 32 | 3.4 | 14 | 37 | IPL |
|  | -18 | -32 | 18 | 3.28 | 7 |  | Thalamus |
|  |  |  |  |  |  |  |  |
| SCO[T2>T1] > CBT[T2>T1] | *Nil* |  |  |  |  |  |  |
|  |  |  |  |  |  |  |  |

Supplementary Table 4. Differences in dorsolateral prefrontal cortex connectivity between baseline (T1) to follow-up (T2) in the cognitive behavioural therapy for psychosis (+CBTp) group compared to standard care only (SCO) groups for direct social threat (angry faces) condition. Voxel threshold of uncorrected p = .001.

| **Contrast** | **MNI coordinates** | | | **Z score** | **Cluster size** | **Brodmann Area** | **Region** |
| --- | --- | --- | --- | --- | --- | --- | --- |
|  | **x** | **y** | **z** |  |  |  |  |
| *+CBTp T1>T2* | *Nil* |  |  |  |  |  |  |
|  |  |  |  |  |  |  |  |
| *+CBTp T2>T1* |  |  |  |  |  |  |  |
|  |  |  |  |  |  |  |  |
|  | -10 | -82 | 24 | 3.62 | 22 | 19/18 | Middle/superior occipital gyrus |
|  |  |  |  |  |  |  |  |
| CBT[T2>T1] > SCO[T2>T1] | -16 | 14 | 34 | 3.56 | 28 | 32 | Anterior cingulate gyrus |
|  | -12 | 38 | 6 | 3.5 | 38 | 11/32 | VMPFC/sgACC |
|  | -10 | 32 | 30 | 3.45 | 24 | 32 | Dorsal anterior cingulate |
|  |  |  |  |  |  |  |  |
|  | 18 | 38 | 24 | 3.42 | 48 | 32 | Dorsal anterior cingulate |
|  | -22 | -34 | 42 | 3.39 | 9 | 31/40 | Posterior cingulate / IPL |
|  | -12 | -48 | 40 | 3.27 | 13 | 31/7 | Posterior cingulate / Precuneus |
|  |  |  |  |  |  |  |  |
| SCO[T2>T1] > CBT[T2>T1] | *Nil* |  |  |  |  |  |  |

Supplementary Table 5. Comparison of healthy participants with psychosis (collapsed across group) and participants in terms of connectivity from amygdala and dorsolateral prefrontal cortex seeds (happy faces condition). Voxel threshold of uncorrected p < .001.

| **Contrast** | **Cluster size** | **MNI coordinates** | | | **Z** | **Brodmann Area** |  |
| --- | --- | --- | --- | --- | --- | --- | --- |
|  |  | **x** | **y** | **z** |  |  | **Region** |
| ***AMYG*** |  |  |  |  |  |  |  |
| *Healthy > Psychosis* | 56 | 30 | -34 | 52 | 3.68 | 3/40 | Somatosensory cortex / IPL |
|  | 16 | 12 | 0 | 40 | 3.49 | 24/23 | dorsal anterior / posterior cingulate  Posterior cingulate |
|  | 24 | -8 | -20 | 46 | 3.49 | 23/4 | Posterior cingulate / motor cortex |
|  | 35 | 42 | -20 | 46 | 3.47 | 4/6 | Motor / premotor cortex |
|  | 20 | -42 | 6 | 26 | 3.35 | 44 | IFG (opercularis) |
|  | 11 | 50 | -16 | 40 | 3.29 | 3/4 | Somatosensory area / motor cortex |
|  | 24 | 14 | -28 | 44 | 3.29 | 23/4 | posterior cingulate / motor cortex |
|  | 13 | 32 | 30 | 32 | 3.25 | 46 | DLPFC |
| *Psychosis > Healthy* | *Nil* |  |  |  |  |  |  |
|  |  |  |  |  |  |  |  |
| ***DLPFC*** |  |  |  |  |  |  |  |
| *Healthy > Psychosis* | 56 | -2 | 2 | 48 | 3.73 | 24 | Dorsal anterior cingulate |
|  | 9 | 24 | -30 | 52 | 3.38 | 3, 4 | Somatosensory area, motor cortex |
|  |  |  |  |  |  |  |  |
| *Psychosis > Healthy* | *Nil* |  |  |  |  |  |  |
|  |  |  |  |  |  |  |  |

Supplementary Table 6. Differences in amygdala connectivity between baseline (T1) to follow-up (T2) in the cognitive behavioural therapy for psychosis (+CBTp) compared to standard care only (SCO) groups for prosocial (happy) facial affect. Voxel threshold of uncorrected p = .001.

| **Contrast** | **Cluster size** | **MNI coordinates** | | | **Z** | **Brodmann Area** | **Region** |
| --- | --- | --- | --- | --- | --- | --- | --- |
|  |  | **x** | **y** | **z** |  |  |  |
| *+CBTp  T1 > T2* |  |  |  |  |  |  |  |
|  | 7 | 16 | -10 | -4 | 3.34 |  | Dorsal striatum |
|  | 7 | 28 | -44 | 26 | 3.28 | 40 | Supramarginal gyrus |
|  |  |  |  |  |  |  |  |
| *+CBTp  T2 > T1* | *Nil* |  |  |  |  |  |  |
|  |  |  |  |  |  |  |  |
| *CBT[T1>T2] > SCO[T1>T2]* | *Nil* |  |  |  |  |  |  |
|  |  |  |  |  |  |  |  |
| *SCO[T2>T1] > CBT[T2>T1]* | *Nil* |  |  |  |  |  |  |

Supplementary Table 7. Differences in dorsolateral prefrontal cortex connectivity between baseline (T1) to follow-up (T2) in the cognitive behavioural therapy for psychosis (+CBTp) compared to standard care only (SCO) groups for prosocial (happy) facial affect. Voxel threshold of uncorrected p = .001.

| **Contrast** | **MNI coordinates** | | | **Z** | **Cluster size** | **Brodmann Area** | **Region** |
| --- | --- | --- | --- | --- | --- | --- | --- |
|  | **x** | **y** | **Z** |  |  |  |  |
| *+CBTp T1 > T2* |  |  |  |  | *Nil* |  |  |
|  |  |  |  |  |  |  |  |
| *+CBTp T2 > T1* |  |  |  |  |  |  |  |
|  | -16 | -28 | -6 | 3.78 | 87 |  | Parahippocampal gyrus |
|  | 28 | -40 | -14 | 3.38 | 10 | 37/36 | Fusiform gyrus |
|  | 16 | -28 | -12 | 3.15 | 5 |  | Parahippocampal gyrus |
| *CBT [T2>T1] > SCO[T2>T1]* | -18 | -26 | 0 | 4.12 | 104 | n/a | Thalamus |
|  | -44 | -20 | 30 | 3.38 | 10 | 3 | Postcentral gyrus |
|  | 62 | -24 | 8 | 3.35 | 9 | 22 | Superior temporal gyrus |

Supplementary Table 8. Associations between significant connectivity and symptom change on the Positive And Negative Symptom Schedule, positive subscale (PANSS-P).

| **PANSS-P item (change)** | ***Anger, Amygdala seed*** | | ***Happy, DLPFC seed*** | |
| --- | --- | --- | --- | --- |
|  | Inferior parietal lobule | Superior parietal lobule | Postcentral gyrus | Thalamus |
| P1 Delusions | 0.306 (0.177) | 0.29 (0.202) | -0.368 (0.101) | ***-0.487 (0.025)*** |
| P2 Disorganisation | 0.097 (0.675) | 0.168 (0.467) | -0.008 (0.972) | -0.031 (0.893) |
| P3 Hallucinations | 0.375 (0.094) | 0.3 (0.186) | ***-0.49 (0.024)*** | -0.409 (0.065) |
| P4 Excitement | -0.087 (0.707) | 0.209 (0.363) | -0.376 (0.093) | -0.227 (0.322) |
| P5 Grandiosity | -0.059 (0.801) | 0.332 (0.141) | -0.238 (0.299) | 0.005 (0.984) |
| P6 Persecution | ***0.473 (0.03)*** | 0.231 (0.314) | -0.221 (0.336) | -0.232 (0.312) |
| P7 Hostility | -0.006 (0.981) | 0.073 (0.753) | -0.139 (0.549) | 0.037 (0.874) |
| Total | 0.384 (0.086) | 0.402 (0.071) | -0.427 (0.054) | -0.394 (0.077) |

Supplementary Table 9. Associations between significant connectivity and symptom change on the Positive And Negative Symptom Schedule, negative subscale (PANSS-N).

| **PANSS-N item (change)** | **Happy, DLPFC seed** | | |
| --- | --- | --- | --- |
|  | Parahippocampal gyrus | Thalamus | Postcentral gyrus |
| N1 Blunted affect | 0.399 (0.073) | 0.387 (0.083) | 0.284 (0.211) |
| N2 Emotional withdrawal | 0.167 (0.469) | 0.125 (0.589) | -0.138 (0.552) |
| N3 Poor rapport | 0.11 (0.636) | 0.07 (0.763) | -0.192 (0.403) |
| N4 Passive social withdrawal | -0.017 (0.942) | -0.118 (0.609) | -0.231 (0.315) |
| N5 Poor abstract thinking | 0.153 (0.508) | -0.098 (0.671) | ***-0.483 (0.027)*** |
| N6 Lack of spontaneity | 0.375 (0.094) | 0.267 (0.242) | 0.275 (0.227) |
| N7 Stereotyped thinking | 0.155 (0.503) | 0.157 (0.496) | -0.046 (0.844) |
| Total | 0.463 (0.035) | 0.499 (0.021) | 0.267 (0.242) |
